# Therapeutic Potential of Regorafenib—A Multikinase Inhibitor in Pulmonary Hypertension

**DOI:** 10.3390/ijms22031502

**Published:** 2021-02-02

**Authors:** Swathi Veeroju, Baktybek Kojonazarov, Astrid Weiss, Hossein Ardeschir Ghofrani, Norbert Weissmann, Friedrich Grimminger, Werner Seeger, Tatyana Novoyatleva, Ralph Theo Schermuly

**Affiliations:** 1 Member of the German Center for Lung Research (DZL), Universities of Giessen and Marburg Lung Center (UGMLC), Excellence Cluster Cardio-Pulmonary Institute (CPI), Justus-Liebig University, 35392 Giessen, Germany; Swathi.Veeroju@innere.med.uni-giessen.de (S.V.); Baktybek.Kojonazarov@innere.med.uni-giessen.de (B.K.); Astrid.Weiss@innere.med.uni-giessen.de (A.W.); Ardeschir.Ghofrani@innere.med.uni-giessen.de (H.A.G.); Norbert.Weissmann@innere.med.uni-giessen.de (N.W.); Friedrich.Grimminger@innere.med.uni-giessen.de (F.G.); Werner.Seeger@innere.med.uni-giessen.de (W.S.); 2 Institute for Lung Health, 35392 Giessen, Germany; 3 Max Planck Institute for Heart and Lung Research, 61231 Bad Nauheim, Germany

**Keywords:** regorafenib (BAY 73-4506), monocrotaline (MCT), chronic hypoxia (HOX), human pulmonary arterial smooth muscle cells, kinome analysis, pulmonary vascular remodeling

## Abstract

Pulmonary hypertension (PH) is characterized by a progressive elevation of mean arterial pressure followed by right ventricular failure and death. Previous studies have indicated that numerous inhibitors of receptor tyrosine kinase signaling could be either beneficial or detrimental for the treatment of PH. Here we investigated the therapeutic potential of the multi-kinase inhibitor regorafenib (BAY 73-4506) for the treatment of PH. A peptide-based kinase activity assay was performed using the PamStation^®^12 platform. The 5-bromo-2′-deoxyuridine proliferation and transwell migration assays were utilized in pulmonary arterial smooth muscle cells (PASMCs). Regorafenib was administered to monocrotaline- and hypoxia-induced PH in rats and mice, respectively. Functional parameters were analyzed by hemodynamic and echocardiographic measurements. The kinase activity assay revealed upregulation of twenty-nine kinases in PASMCs from patients with idiopathic PAH (IPAH), of which fifteen were established as potential targets of regorafenib. Regorafenib showed strong anti-proliferative and anti-migratory effects in IPAH-PASMCs compared to the control PASMCs. Both experimental models indicated improved cardiac function and reduced pulmonary vascular remodeling upon regorafenib treatment. In lungs from monocrotaline (MCT) rats, regorafenib reduced the phosphorylation of c-Jun N-terminal kinase and extracellular signal-regulated kinase 1/2. Overall, our data indicated that regorafenib plays a beneficial role in experimental PH.

## 1. Introduction

Pulmonary arterial hypertension (PAH) is a progressive disease with an increase in mean pulmonary arterial pressure ≥25 mm Hg and right ventricular hypertrophy followed by a decline in heart function and failure [1,2]. The elevated pressure in the pulmonary circulation is mainly due to pathological remodeling of the pulmonary vasculature [3,4,5,6]. Though the therapies for increasing the survival span of PH patients exist, an optimal outcome is yet to be achieved [7]. Recent advances in understanding the molecular mechanism of disease progression demonstrate an overactivation of various kinases, offering them as potential therapeutic targets [8,9,10].

Both receptor tyrosine kinases (RTKs) and non-receptor tyrosine kinases (non-RTKs) play a remarkable role in the pathophysiology of PH affecting numerous cellular processes like proliferation, migration, apoptosis, metabolism, and cell survival. These abnormal cellular changes are stimulated by various growth factors like epidermal growth factors (EGFs), fibroblast growth factors (FGFs), and platelet-derived growth factors (PDGFs), which trigger kinase-dependent signaling pathways in PH [3,7,11,12,13,14].

Similar to cancer, an increased expression and activity of various kinases is a characteristic hallmark of PH [14]. Various kinases, including TKs, modulate the intracellular signals that activate survival, proliferation, and migration of cells, implying their role as drivers for both pseudo-malignant PH and cancer. In addition, many growth factors and receptors work in a complementary and coordinated manner in both diseases. Thus, targeting various kinases with multi-kinase inhibitors (MKIs) or tyrosine kinase inhibitors (TKIs) approved for cancer therapy has great importance in the advancement of novel therapeutics in PH [11,14,15]. Most of the kinase inhibitors are small-molecule inhibitors that prevent the activation of phosphorylation by blocking the adenosine triphosphate (ATP) binding region in the kinase domain of RTKs and non-RTKs [3,14]. Treatment with TKIs like imatinib (Bcr-Abelson tyrosine-protein kinase (Abl), c-Kit, PDGFR-β antagonist) [13,15], nilotinib (Bcr-Abl, c-Kit, PDGFR-α/β inhibitor), dasatinib (Bcr-Abl, c-Kit, PDGFR-α/β, and Src inhibitor) [16,17], EGFR blockers like erlotinib, gefitinib, lapatinib [18], and MKIs like sunitinib (PDGFR-β, VEGFR, c-KIT, FLT3) or sorafenib (b-RAF, c-Kit, FLT3, PDGFR-β, RAF-1, VEGFR-2/3) were reported in pre-clinical studies. Although some clinical studies with these inhibitors have shown improved pulmonary and cardiac function (in particular using imatinib), none of these inhibitors are currently approved for PH therapy due to unfavorable side effects [13,14,17,19,20].

Regorafenib (BAY 73-4506) is a multi-kinase inhibitor, approved by the US Food and Drug Administration (FDA) for the treatment of advanced gastrointestinal stromal tumor (GIST) and colorectal carcinoma (CRC) [21]. Regorafenib is also approved by the FDA as second-line therapy for hepatocellular carcinoma (HCC) in patients treated with sorafenib [22,23,24,25,26]. Regorafenib targets the activity of various kinases, including PDGFR-β, FGFR 1, vascular endothelial cell-derived growth factor receptors 1–3 (VEGFR 1–3), TEK receptor tyrosine kinase-2 (TIE2), discoidin domain receptor tyrosine kinase 2 (DDR2), ephrin type-A receptor 2 (Eph2A), proto-oncogenes like RET, KIT, C-RAF, B-RAF, protein tyrosine kinase 5 (PTK5), Abl, and mitogen-activated protein kinases (MAPKs), including stress-activated protein kinases/c-Jun N-terminal kinases (SAPK/JNK) and p38 MAPK. The inhibition profile of these kinases by regorafenib was determined by in vitro biochemical assays [27]. In vitro and in vivo preclinical studies utilizing various cancer cell lines and cancer models, as well as clinical investigations established the anti-proliferative, anti-migratory, anti-angiogenic, and anti-cancer effects of regorafenib [26,28,29,30]. The objective of this study was to determine if regorafenib would have any impact on cardiac function and pulmonary vascular remodeling in experimental PH.

## 2. Results

### 2.1. Effect of Regorafenib on Kinase Activity in Pulmonary Smooth Muscle Cells Isolated from Patients with Idiopathic Pulmonary Arterial Hypertension

**Figure 1:**
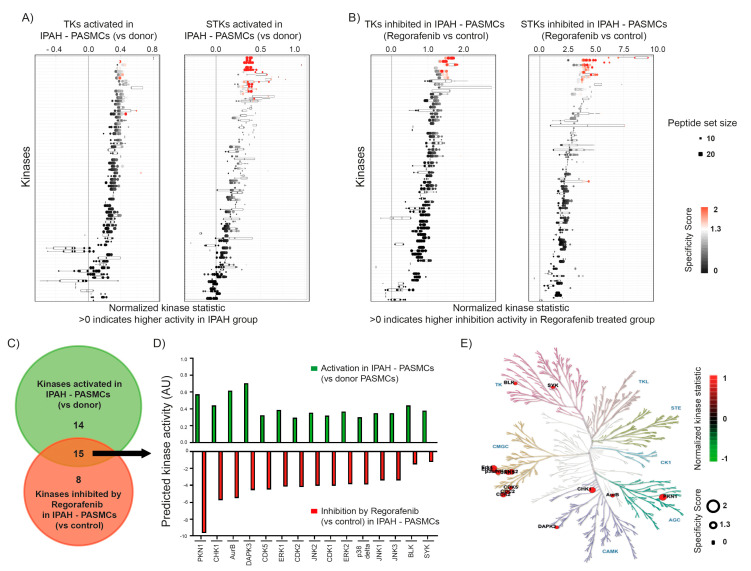
Effect of regorafenib on tyrosine kinases (TKs) and serine/threonine kinases (STKs) in idiopathic pulmonary arterial hypertension smooth muscle cells. Kinome analysis of tyrosine kinases (TKs) and serine/threonine (STKs) kinases carried out by peptide array-based kinase activity assay in donor and idiopathic pulmonary arterial hypertension (IPAH)-pulmonary arterial smooth muscle cells (PASMCs) (*n* = 3, biological replicates). Normalized statistic kinases representing predicted kinase activity of TKs and STKs (**A**) (>0) in IPAH versus donor PASMCs and (**B**) expected inhibition (>0) by regorafenib treatment against DMSO control in IPAH-PASMCs. *Y*-axis shows the list of kinases while the size of dots indicates the respective peptide set size (10 to 20). The number of dots for each kinase is an outcome of an individual permutation analysis of peptides with varying rank-cut off values. Specificity score (0–2) indicates statistical relevance, >1.3 (negative log10 (*p*-value) with *p* = 0.05) is statistically significant. (**C**) Venn diagram represents differentially regulated kinases in IPAH-PASMCs (29, highlighted in green) and regorafenib treated IPAH-PASMCs (23, highlighted in red) as well as an intersecting set of candidates. (**D**) The graph representing 15 overlapping kinases (on *X*-axis) activated in IPAH-PASMCs (in green) and their inhibitory pattern by regorafenib treatment (in red), AU: Arbitrary units. (**E**) Representation of 15 kinases (regorafenib-mediated inhibition in IPAH-PASMCs) in the phylogenetic kinome tree containing the following groups: TK (tyrosine kinase), TKL (tyrosine kinase-like), AGC (protein kinase A, G, C families), CAMK (calcium/calmodulin-dependent protein kinase), CK1 (casein kinase 1), CMGC (CDK, MAPK, GSK3, CLK families) and STE (homologs of yeast Sterile 7, Sterile 11, Sterile 20 kinases). Normalized kinase activity with (>0, black to red) indicates significant kinase inhibition with regorafenib treatment.

To investigate the effect of regorafenib on tyrosine kinase (TK) and serine/threonine kinase (STK) activity, a peptide-based kinase activity screening was performed. Upstream kinases that are responsible for these observed alterations are predicted using bioinformatics tools. In the first step, we compared the activity of kinases between donor pulmonary arterial smooth muscle cells (donor PASMCs) and PASMCs from patients with idiopathic PAH (IPAH-PASMCs). This technology has recently been described by our group in the context of PAH [9,31]. As shown in Figure 1A, IPAH PASMCs exhibit increased activity of both tyrosine kinases (TKs) and serine/threonine kinases (STKs), among which twenty-nine kinases were altered for both kinase classes (Supplemental Table S1). In the next step, we compared the activity of the dimethyl sulfoxide (DMSO) control IPAH-PASMCs with regorafenib-treated IPAH-PASMCs where the latter ones displayed a high inhibitory activity induced by regorafenib, as indicated in Figure 1B. From both TKs and STKs, twenty-three kinases were inhibited by regorafenib treatment in IPAH-PASMCs (Supplemental Table S2). Here, the DMSO control and regorafenib treatment-specific signatures of the peptide-phosphorylation pattern due to distinct kinase activities were highlighted, as represented in the corresponding heat map (Figure S1). Next, we aimed to estimate the impact of regorafenib on those kinases, where activity has shown to be augmented in IPAH-PASMCs in comparison to donor PASMCs. While twenty-nine kinases were activated in IPAH-PASMCs in comparison to donor PASMCs, twenty-three kinases were inhibited by regorafenib in IPAH-PASMCs. Venn diagram analysis demonstrated an intersecting set of TKs and STKs that were affected in both types of comparisons namely upregulated in donor versus IPAH-PASMCs and IPAH-PASMCs with or without regorafenib treatment (Figure 1C). Notably, fifteen out of twenty-nine kinases activated in IPAH-PASMCs were inhibited by regorafenib, indicating its multi-kinase inhibitor profile (Figure 1D). The distribution of these fifteen kinases within the phylogenetic tree indicated that they belong to one of the following kinome families: TK (tyrosine kinase) family, AGC family includes cAMP-dependent protein kinase (PKA), cGMP-dependent protein kinase (PKG), protein kinase C (PKC), calcium/calmodulin-dependent protein kinase (CAMK) family, or the CMGC family including cyclin dependent kinases (CDK), mitogen-activated protein kinase (MAPK), glycogen synthase kinase (GSK), and CDK-like kinase (CLK) (Figure 1E).

### 2.2. Regorafenib Inhibits Proliferation and Migration of Human Pulmonary Arterial Smooth Muscle Cells

**Figure 2:**
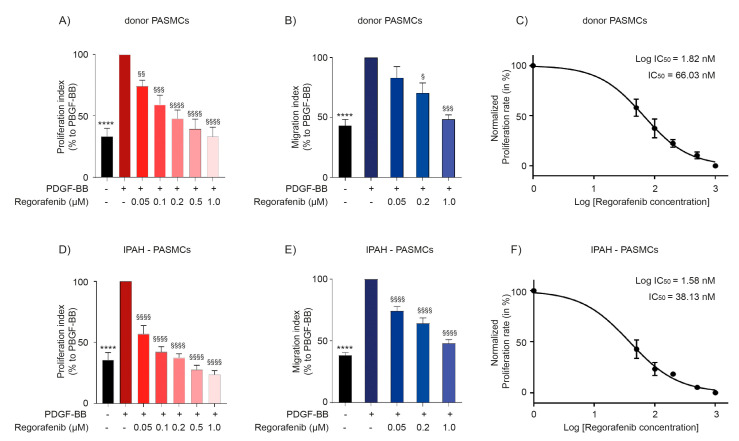
Effect of regorafenib on proliferation and migration of human pulmonary arterial smooth muscle cells. Proliferation index measured by BrdU incorporation assay in (**A**) donor PASMCs (*n* = 6) and (**D**) IPAH-PASMCs (*n* = 6) stimulated with PDGF-BB followed by treatment with regorafenib in a dose-dependent manner. Migration index measured by transwell migration assay in (**B**) donor (*n* = 3) and (**E**) IPAH (*n* = 4) PASMCs stimulated with PDGF-BB followed by treatment with regorafenib in a dose-dependent manner. IC_50_ values were calculated from proliferation rate in (**C**) donor and (**F**) IPAH-PASMCs. **** *p* < 0.0001, versus DMSO control; ^§§§§^ *p* < 0.0001, ^§§§^ *p* < 0.001, ^§§^ *p* < 0.01, and ^§^ *p* < 0.05 versus PDGF-BB treatment (30 ng/mL). Data represent the mean ± SEM.

To investigate the effect of regorafenib on proliferation and migration, human PASMCs from donor controls and patients with IPAH pre-treated with regorafenib were stimulated with the platelet-derived growth factor (PDGF-BB). Proliferation was assessed by BrdU (5-bromo-2′-deoxyuridine) incorporation assay and migration rates were estimated by employing the transwell chamber assay. Importantly, regorafenib exhibited strong anti-proliferative (Figure 2A,D) and anti-migratory activities (Figure 2B,E) in PASMCs in a concentration-dependent manner. Half-maximal inhibitory concentration (IC_50_) calculated from proliferation verified that regorafenib has remarkable inhibitory activity on IPAH-PASMCs with an IC_50_ of 38.13 and 66.03 nM in donor PASMCs (Figure 2C,F).

### 2.3. Regorafenib Improves Cardiac Function and Reverses Pulmonary Vascular Remodeling Both in Hypoxia- and Monocrotaline-Induced Pulmonary Hypertension

**Figure 3:**
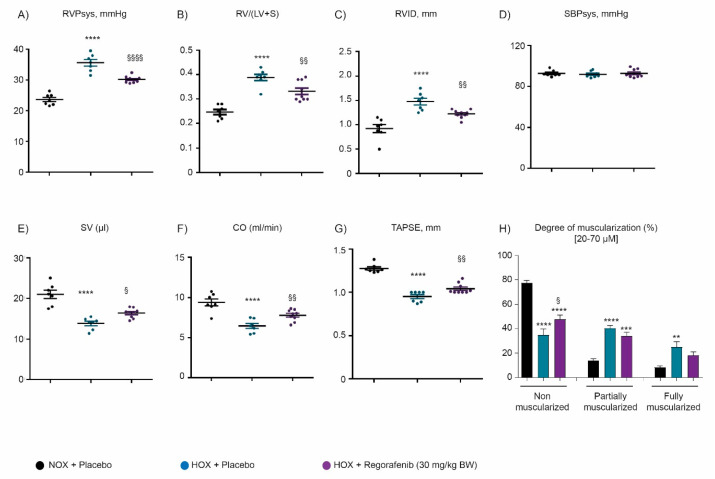
Effect of regorafenib on cardio-pulmonary functions in chronic hypoxia (HOX)-induced pulmonary hypertension. (**A**) Right ventricular systolic pressure (RVSP, mmHg). (**B**) Fulton index measured as the ratio of RV to LV plus septum (RV/LV + S). (**C**) Right ventricle internal diameter (RVID, mm). (**D**) Systemic arterial pressure (SBPsys, mmHg). (**E**) Stroke volume (SV, µL). (**F**) Cardiac output (CO, mL/min). (**G**) Tricuspid annular plane systolic excursion (TAPSE, mm) and (**H**) degree of muscularization was measured as a percentage of small pulmonary arteries (20–70 µM) characterized by non-muscularized, partially muscularized, and fully muscularized in NOX + placebo (*n* = 7), HOX + placebo (*n* = 7), and HOX mice treated with regorafenib (*n* = 9). **** *p* < 0.0001, *** *p* < 0.001, and ** *p* < 0.01 versus NOX + placebo; ^§§§§^ *p* < 0.0001, ^§§^ *p* < 0.01 and ^§^ *p* < 0.05 versus HOX + placebo. Data represent the mean ± SEM.

**Figure 4:**
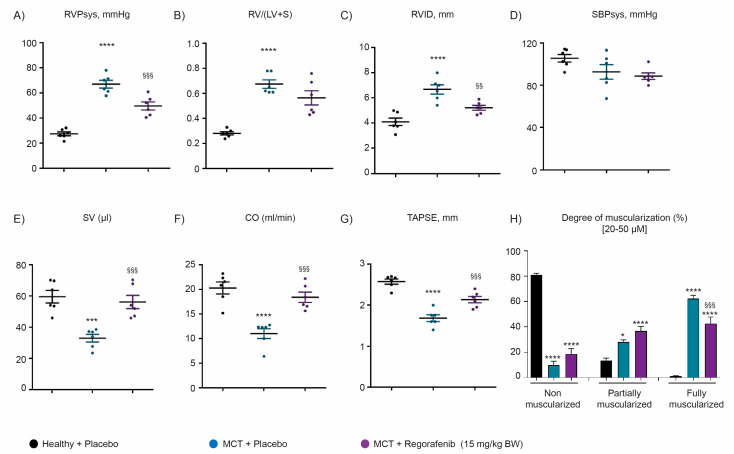
Effect of regorafenib on cardio-pulmonary functions in monocrotaline (MCT)-induced pulmonary hypertension. (**A**) Right ventricular systolic pressure (RVSP, mmHg). (**B**) Fulton index measured as the ratio of RV to LV plus septum (RV/LV + S). (**C**) Right ventricle internal diameter (RVID, mm). (**D**) Systemic arterial pressure (SBPsys, mmHg). (**E**) Stroke volume (SV, µL). (**F**) Cardiac output (CO, mL/min) (**G**) Tricuspid annular plane systolic excursion (TAPSE, mm), and (**H**) Degree of muscularization was measured as a percentage of small pulmonary arteries (20–50 µM) characterized by non-muscularized, partially muscularized, and fully muscularized in healthy + placebo (*n* = 6), MCT + placebo (*n* = 4–6) and MCT rats treated with regorafenib (*n* = 6). **** *p* < 0.0001, *** *p* < 0.001, and * *p* < 0.05 versus healthy + placebo; ^§§§^ *p* < 0.0001, and ^§§^ *p* < 0.01 versus MCT + placebo. Data represent the mean ± SEM.

To investigate the effect of a regorafenib therapy in experimental PH, a chronic-hypoxia induced mouse model of PH (HOX) and monocrotaline-induced rat model of PH (MCT) were investigated. Both chronic hypoxia and MCT treatment led to a significant increase in right ventricular systolic pressure (RVSP, mmHg) (Figure 3A and Figure 4A), right ventricular internal diameter (RVID, mm) (Figure 3C and Figure 4C), and right ventricular hypertrophy (RV/LV + S) (Figure 3B and Figure 4B), confirming the occurrence of the PH. An increase of RV hypertrophy was tightly associated with a decrease in stroke volume (SV) (Figure 3E and Figure 4E), cardiac output (CO) (Figure 3F and Figure 4F), and tricuspid annular plane systolic excursion (TAPSE) parameters (Figure 3G and Figure 4G) in both models of PH.

Therapeutic administration of regorafenib from days 21 to 35 improved the hemodynamics with a significant reduction in RVSP, RVID, and RV/LV + S (Figure 3A–C and Figure 4A–C) in both experimental models of PH. Concomitantly with attenuation of hemodynamics, regorafenib treatment ameliorated cardiac function, as accessed by an increase in SV, CO, and TAPSE echocardiography parameters in both rodent models of PH (Figure 3E–G and Figure 4E–G). Regorafenib treatment did not affect systemic arterial pressure (SBPsys, mmHg) in both experimental models of PH (Figure 3D and Figure 4D). Furthermore, regorafenib improved pulmonary vascular remodeling, as demonstrated by significant elevation of non-muscularized small pulmonary arteries (Figure 3H) and a reduction in fully muscularized small pulmonary arteries (Figure 4H) in HOX-induced and MCT-treated animals, respectively.

### 2.4. Regorafenib Attenuates the Activity of MAPK Signaling via ERK and JNK

**Figure 5:**
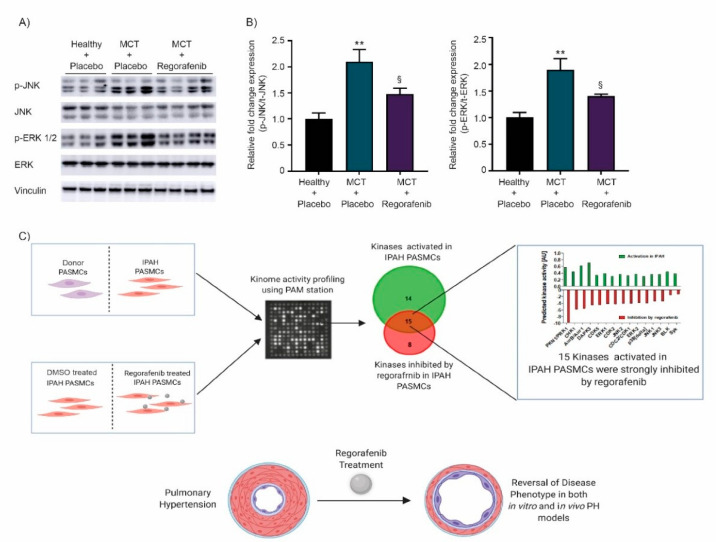
Effect of regorafenib on c-Jun N-terminal kinase (JNK) and extracellular signal-regulated kinases (ERK) and mitogen-activated protein kinases (MAPK) signaling in the MCT model. (**A**) Western blots representing p-JNK, t-JNK, p-ERK1/2, and t-ERK. Vinculin serves as a loading control. (**B**) Densitometric analysis showing relative fold activity of p-JNK/t-JNK and p-ERK/t-ERK (*n* = 3–4 per group). ** *p* < 0.01 versus healthy + placebo; § *p* < 0.05 versus MCT + placebo. Data represent the mean ± SEM. (**C**) Schematic representation of the regorafenib mode of action in PH (created with BioRender.com).

Kinome analysis in human pulmonary arterial smooth muscle cells (hPASMCs) demonstrated activation of mitogen-activated protein kinases (MAPKs), such as c-Jun N-terminal kinase (JNK) and extracellular signal-regulated kinase 1/2 (ERK 1/2) in IPAH-PASMCs, previously reported to contribute to PH progression and vascular remodeling [14,32,33]. As treatment with regorafenib inhibited activity of ERK and JNK/MAPK signaling in IPAH-PASMCs, we aimed to verify their activity by measuring the phosphorylation level by western blotting of protein extracts from lungs from MCT-treated rats. The MCT-induced increase in phosphorylation of ERK 1/2 and JNK was significantly attenuated by regorafenib treatment (Figure 5A,B), suggesting that regorafenib beneficial effects on cardiac function and pulmonary vascular remodeling are mediated by inhibition of ERK and JNK/MAPK signaling pathways.

## 3. Discussion

Employing a peptide-based kinase activity assay platform, we identified that the activity of several kinases, as tyrosine kinases (TKs) and serine/threonine kinases (STKs) were markedly augmented in PAH-PASMCs. Interestingly, more than half of the kinases activated in PAH-PASMCs were inhibited by treatment with multi-kinase inhibitor regorafenib. Furthermore, we demonstrated that regorafenib exhibits strong anti-proliferative and anti-migratory effects in human PASMCs from both donor individuals and patients with IPAH, providing stronger effects on diseased PASMCs. These findings were correlated with the data establishing regorafenib as a therapeutic compound for the treatment of both hypoxia- and non-hypoxia-induced PH.

Multiple lines of evidence indicate that numerous signaling pathways, dysregulated in PH, are commonly driven by the activation of various TKs and STKs. Previous reports show that targeting of these kinases with small molecular tyrosine or multi-kinase inhibitors (TKIs or MKIs) provide functional improvement in experimental pre-clinical PH models and clinical studies [3,7,34]. The TKI imatinib (Abl, kit, PDGFR-β antagonist) has demonstrated functional improvement in experimental PH [13]. Other TKIs like erlotinib, gefitinib, lapatinib, which target epidermal growth factor receptor (EGFR), revealed beneficial effects on cardiac function and vascular remodeling in experimental PH [14,18]. MKIs sorafenib or sunitinib, investigated in pre-clinical PH models, provided improved hemodynamics and remodeling [20,35,36]. Although in experimental PH, various TKIs and MKIs exhibited beneficial impact on the disease progression, a large range of inhibition and lack of specificity were postulated to cause unanticipated toxicity in various organs [3].

Though in clinical studies imatinib improved hemodynamic parameters in patients with advanced PAH, providing a positive response [37,38,39,40,41,42], the drug was not approved for PAH therapy due to some adverse side events such as vomiting, peripheral edema, and subdural hematomas. A clinical study conducted with sorafenib for safety and tolerability in advanced stage PAH patients imposed negative effects on cardiac function, whereas a clinical study with a small group of refractory PAH patients exhibited tolerable side-effects with improved hemodynamic function, although few patients were discontinued from the study due to adverse side effects [43,44]. This raises the question of whether PH-tailored TKI like regorafenib might represent a new and hopeful treatment for PH.

In this study, peptide-based screening revealed activation of both TKs and STKs in IPAH-PASMCs compared to donor PASMCs inconsistent with previous reports from our group [9,31]. Regorafenib is an oral multi-targeted tyrosine kinase inhibitor, which inhibits angiogenic, and oncogenic receptor tyrosine kinases, as well as the RAF/MEK/ERK signaling pathway in cancer [29,45,46]. Treatment with regorafenib in human PASMCs from IPAH patients revealed a substantial kinase inhibitory pattern by PamGene analysis. Interestingly, among twenty-nine kinases activated in IPAH-PASMCs, fifteen kinases were inhibited by regorafenib treatment. Among them, several mitogen-activated protein kinases (MAPKs), including extracellular signal-regulated kinases (ERK1 and ERK2) [47,48], JUN N-terminal kinases (JNK1, JNK2, and JNK3) [49,50,51], and p38 delta [32,33,50,52] were reported to contribute to PAH pathophysiology. Additionally, the regorafenib mediated inhibition of cell cycle regulatory kinases, which play a pivotal role in disease progression, as checkpoint kinase 1 (Chk1) [53,54], cyclin-dependent kinases (CDK1 and CDK2) [3,14,31] were noticed. Interestingly, many of these kinases, including death-associated protein kinase 3 (DAPK3) [55,56,57] were previously investigated in the PH context, while targeting of these kinases provided beneficial impacts on cardio-pulmonary functions in experimental PH [3,14]. Aurora B kinase (AurB) is a serine/threonine kinase important for cell cycle regulation and mitosis. It is highly expressed in various tumors including breast, colorectal, kidney, prostate, non-small cell lung carcinoma, KRAS-induced lung cancer, and targeting AurB kinases displayed inhibition of cancer progression [58,59,60,61,62]. Furthermore, inhibition of growth factors like forkhead box M1 (FOXM1) and polo-like kinase 1 (PLK1) in PAH-PASMCs displayed a significant reduction of cell cycle component AurB [63], suggesting that targeting AurB kinase might have beneficial effects in treating PH. Inhibition of protein kinase N1 (PKN1) has shown anti-proliferative and anti-migratory effects in aortic vascular smooth muscle cells and vascular remodeling in the carotid artery balloon injury (BI) model [64]. Spleen-associated tyrosine kinase (SYK) inhibitor (BAY61-3606) treatment decreased the growth factor-induced proliferation and migration in vascular smooth muscle cells [65]. Studies have shown that the active form of human B lymphocyte kinase (BLK) is a proto-oncogene and targeting it using an inhibitor has shown anti-proliferative effect in cutaneous T-cell lymphoma (CTCL) [66]. These studies indicate that AurB, PKN1, BLK, and SYK kinases have pro-proliferative and pro-migratory characteristics, the hallmark of PH pathogenesis. It will be interesting to further investigate the direct role of these kinases in PH.

Pathological remodeling of the pulmonary vasculature in PH is driven by pro-proliferative and pro-migratory characteristics of the vascular cells [3,13,67]. Pro-proliferation and resistance to apoptosis are common characteristic phenotypes of cancer and PAH. Regorafenib reported inhibiting the proliferation and migration of multiple cancer cells [3,24,28,29,68]. We observed that treatment with regorafenib exhibited strong anti-proliferative and anti-migratory effects in IPAH derived PASMCs, compared to donor PASMCs, indicating that IPAH-cells are more susceptible to the treatment with this MKI.

To investigate the effect of regorafenib on experimental PH, two independent experimental models of PH, chronic hypoxia (HOX)- and monocrotaline (MCT)-induced PH, were employed. In both models, treatment with regorafenib exhibited improved function with reduced right ventricular systolic pressure (RVSP), followed by significant improvements in cardiac output (CO), stroke volume (SV) and tricuspid annular plane systolic excursion (TAPSE). The histomorphometric analysis of pulmonary vascular remodeling demonstrated a decrease in the degree of muscularization with a significant reduction in fully muscularized vessels in MCT-treated rats and augmentation of non-muscularized vessels in hypoxic mice, suggesting that regorafenib can act as anti-remodeling therapy for PH.

Accumulating data evidence that activation of MAPK signaling kinases, including ERK and JNK occur in experimental PH and the reduction in the activity of these kinases follows the therapeutic targeting of PH [14,33,48,49,51]. In line with this, lung homogenates obtained from regorafenib treated MCT rats showed reduced phosphorylation of ERK and JNK comparable to their activity in regorafenib treated IPAH-PASMCs accessed from PamGene analysis. A limitation of the current study is that the effects of regorafenib have not been determined in the healthy-control group of animals. Multiple lines of evidence indicate that regorafenib induces life-threatening cardiotoxicity including hypertension and cardiac ischemia/infarction, the effects, which may be related to mitochondrial damage [69]. Thus, regorafenib pro-longed administration may cause cardiotoxic effects, which ultimately may result in an impairment of RV function. Two case reports demonstrated that regorafenib induces myocardial injury during atrial fibrillation in a 72-year old patient with metastatic GIST [70], and congestive heart failure in a 13-year-old patient with recurrent osteosarcoma [71]. Cardiovascular toxicity of regorafenib has been also described for patients with solid tumors [72]. Moreover, toxicity and intolerability of this TKI lead to discontinuation of regorafenib treatment, because of intolerability in 25.6% of patients with advanced stage CRC, HCC, and GIST [73]. Thus, adequate awareness of cardiovascular adverse events and potential cardiac dysfunction of regorafenib in future is warranted. In conclusion, our data suggest that multi-kinase inhibitor regorafenib, although with great precautions, can be considered either as a novel therapeutic option or as a potential adjuvant for pre-existing PH/PAH medications.

In conclusion, our data suggest that multi-kinase inhibitor regorafenib, although with great precautions, can be considered either as a novel therapeutic option or as a potential adjuvant for pre-existing PH/PAH medications.

## 4. Materials and Methods

### 4.1. Drugs and Antibodies

Regorafenib (BAY 73-4506) and dimethyl sulfoxide (DMSO) were purchased from Selleckchem (Houston, TX, USA) and Sigma–Aldrich (St. Louis, MO, USA). Platelet-derived growth factor-beta (PDGF-BB) was purchased from R&D Systems (Minneapolis, MN, USA). Crystal violet and 4′,6′-diamidino-2-phenylindole (DAPI) were from Sigma–Aldrich (Munich, Germany). The following antibodies were used in this study: Phospho-ERK 1/2 (Thr 202/Tyr 204), ERK (Santa Cruz Biotechnology, Dallas, TX, USA), Phospho-SAPK/JNK (Thr183/Tyr185), SAPK/JNK (Cell Signaling, Danvers, MA, USA), von Willebrand factor (vWF, Dako, Hamburg, Germany), alpha smooth muscle actin (α-SMA, Sigma–Aldrich, Munich, Germany) and vinculin (Sigma–Aldrich, MO, USA).

### 4.2. In Vivo Experiments

All in vivo experiments were approved by the local authorities (Regierungspräsidium, Giessen, Germany, approval no. GI20/10 Nr. G50/2016). The monocrotaline (MCT) model of PH was performed in Sprague–Dawley (SD) rats (300–350 g body weight) by subcutaneous injection of 60 mg of MCT per kg body weight. After three weeks, regorafenib (15 mg per kg body weight) or a placebo (1% methylcellulose) was administered every day by oral gavage for another two weeks. Administration of saline served as a healthy control group. In the chronic hypoxia-induced PH (HOX) model, C57BL/6J mice (21–24 g body weight) were exposed to hypoxia (with 10% oxygen) for five weeks. After 3 weeks of hypoxia, mice were treated with regorafenib (30 mg per kg of body weight) or placebo (1% methylcellulose) every day by oral gavage for the following 2 weeks of hypoxic exposure. A healthy control group was maintained at normoxic (NOX) conditions. After five weeks, functional parameters were accessed from both the models and processed for organ isolations. Hemodynamic measurements were performed to obtain right ventricular systolic pressure (RVPsys, mmHg) and systemic arterial pressure (SBPsys, mmHg), and right ventricular hypertrophy was measured by calculating the ratio of the right ventricle (RV) to left ventricle (LV) plus septum (S) (RV/LV + S) [71]. Echocardiography measurements were executed by non-invasive transthoracic imaging to retrieve cardiac functions like stroke volume (SV, µL), cardiac output (CO, mL/min), right ventricular internal diameter (RVID, mm), and tricuspid annular plane systolic excursion (TAPSE, mm) [31,52,74,75,76,77]. Vascular remodeling was evaluated by the degree of muscularization (in percentage) measured from double staining of alpha-smooth muscle actin (α-SMA) and von Willebrand factor (vWF). Vessels with a diameter of 20–70 (for mice) and 20–50 μM (for rats) were analyzed and categorized as non-muscularized (<5% smooth muscle actin (SMA) around the vessel), partially muscularized (5–75% SMA around the vessel) or fully muscularized (≥75% SMA around the vessel) [18,76,77].

### 4.3. Cell Culture Experiments

Human pulmonary arterial smooth muscle cells (hPASMCs) isolated from healthy individuals without PAH (donors) were purchased from Lonza (Basel, Switzerland) and PASMCs from idiopathic pulmonary arterial hypertension (IPAH) patients were obtained from Giessen biobank, Justus–Liebig University, Germany. The collection of the human material was performed in accordance with the protocol approved by the ethics committee of the University Hospital Giessen (Germany) in accordance with the European IPS registry (AZ 111/08) and DZL Biobank (58/15). Tissue donation was approved by the ethics committee of the University Hospital Giessen in agreement with the general principles in the Declaration of Helsinki.

Cells were cultured in growth medium smooth muscle cell growth medium (SMGM) containing a cocktail of insulin, human fibroblast growth factor (hFGF), fetal bovine serum (FBS), and human epidermal growth factor (hEGF) obtained from Lonza (Basel, Switzerland). All experiments were performed between passages three to five in the starvation medium (SMBM, without the addition of growth factors) (Basel, Switzerland) or SMGM with or without PDGF-BB stimulation (30 ng/mL).

### 4.4. Proliferation Assays

Proliferation assays were performed in both donor PASMCs and IPAH-PASMCs by 5-bromo-2′-deoxyuridine (BrdU) incorporation assay (Roche Diagnostics, Basel, Switzerland) as per manufacturer’s instructions. Briefly, cells were split into a 96-well plate with 4000 cells per well. The next day, cells were replaced with smooth muscle cell basal medium (SMBM). After 24 h of starvation, cells were pretreated with regorafenib at various concentrations (0.05, 0.1, 0.2, 0.5, and 1 µM) for 90 min and then stimulated with PDGF-BB for up to 24 h. Treatment with dimethyl sulfoxide (DMSO) served as a control. Cells were incubated with BrdU for 4 h prior to the assay and the effect of the compound on proliferation was measured 24 h after the treatment using a spectrophotometer at 370 nM absorbance.

### 4.5. Migration Assays

Migration assays were performed in both donor and IPAH-PASMCs by transwell chamber assay (Corning Life Sciences, Taufkirchen, Germany), as described previously [77]. Briefly, 24-h post-starvation, 12,000 cells per well (regorafenib treated or DMSO control) were seeded onto the upper part of the transwell inserts and placed in SMBM. After 90 min, the media in the lower part was stimulated with PDGF-BB for another 16 h during which cells migrated from the upper side of the membrane to the lower side. Meanwhile, PDGF-BB stimulation-induced migration of cells from the upper side to the lower side of the transwell membrane. Next day, the medium was aspirated from the upper part of the transwell and gently cleaned with a cotton swab. Cells migrated on the lower part were fixed with 3.7% paraformaldehyde (PFA) in 1X phosphate-buffered saline (PBS) for 10 min and afterward washed with 1X PBS for 5 min. Cells were then stained with 0.1% crystal violet (in methanol) for 15 min, followed by DAPI counterstaining for another 5 min to obtain nuclear staining. Anti-migratory effects were analyzed under a fluorescence microscope by counting the number of DAPI positive cells in five different regions per condition.

### 4.6. Kinome Analysis

Donor and IPAH-PASMCs were cultured in 10-cm dishes until they reach 60–70% confluency and starved for 24 h in a starvation medium (SMBM). The next day, cells were treated with either dimethyl sulfoxide (DMSO) as a control, or regorafenib (10 nM) for 90 min and then stimulated with SMGM including PDGF-BB for 15 min. Media was aspirated and cells were washed with ice-cold PBS and then harvested with M-PER lysis buffer (Thermo Fisher Scientific, Waltham, MA, USA) containing a protease and phosphatase inhibitor cocktail (Pierce, Rockford, IL, USA). The lysate collected was passed through a syringe 3–4 times for mechanical disrupture prior to protein isolation. Lysates were incubated for 1 h at 4 °C under constant rotation and centrifuged at 4 °C with 16,000 *g* for 15 min. The supernatant was distributed into 15 µL aliquots and stored at −80 °C until utilized for further analysis. Kinase activity measurement was performed using the PamStation^®^12 platform (PamGene International, s-Hertogenbosch, the Netherlands) with Evolve 12 software. Description of the peptide-based activity assay and its analysis have been explained in detail previously [9,31,78,79].

### 4.7. Western Blot Analysis

Total protein was isolated from total lung homogenates from MCT model were isolated using cell lysis buffer (Cell Signaling Technology, MA, USA). Protein normalized were passed through NuPAGE Bis-Tris gels (Thermo Fisher Scientific, Waltham, MA, USA). Proteins separated were blotted on nitrocellulose membrane and blocked with 5% milk (in 1X TBS/T) for 1 h at room temperature (RT). All the primary antibodies were diluted in 5% BSA (in 1XTBS/T) and were incubated overnight at 4 °C. The next day, membranes were washed with 1X TBS/T 3 times followed with incubation of horseradish peroxidase (HRP) conjugated secondary antibody (in 5% milk) for 1 h, RT. Membranes were then washed another 3 times, 15 min each with 1XTBS/T. The protein expression was detected by chemiluminescence using ECL Prime western blotting detection reagent (GE Healthcare GmbH, Solingen, Germany).

### 4.8. Statistical Analysis

Statistical analysis was performed using GraphPad Prism 6.0 software (GraphPad, San Diego, CA, USA). Multiple comparisons were analyzed by one-way ANOVA with a Newman–Keuls post-hoc test and Student′s *t*-test for analysis between two groups. Half maximal inhibitory concentration (IC_50_) values were calculated from the relative fold proliferation rate using GraphPad prism software. *p*-values ˂ 0.05 were considered to be statistically significant and data were expressed as mean ± SEM.

## Data Availability

All data reported in the manuscript and in the Supplementary Material.

## Supplementary Materials

The following are available online at https://www.mdpi.com/1422-0067/22/3/1502/s1, Figure S1: Expression pattern of regorafenib treatment on substrate peptide phosphorylation in idiopathic pulmonary arterial hypertension (IPAH) PASMCs, Table S1: List of TKs and STKs upregulated in IPAH-PASMCs, Table S2: Kinases inhibited by regorafenib treatment in IPAH-PASMCs.
