# Supplementary material for: Therapeutic Potential of Regorafenib—A Multikinase Inhibitor in Pulmonary Hypertension
Source: Int J Mol Sci. 2021 Feb 2;22(3):1502. doi: 10.3390/ijms22031502 (PMC7867319; doi:10.3390/ijms22031502)
Supplement: Supplementary file 1 [file ijms-22-01502-s001.pdf]

Regorafenib- a novel therapeutic approach for the treatment of experimental Pulmonary Hypertension

Supplemental tables:

Table 1. List of TKs and STKs upregulated in IPAH-PASMCs (compared to donor-PASMCs)

Type	Kinase Uniprot ID	Kinase Name	Mean Specificity Score	Median Kinase Statistic
PTK	Q08345	DDR1	1,516460479	0,800631998
PTK	P43405	Syk	1,392595087	0,379270616
PTK	P42680	TEC	1,099043536	0,417297439
PTK	P51451	BLK	1,06061171	0,440685869
PTK	P43403	ZAP70	1,02124757	0,372014803
PTK	P30530	Axl	1,009309564	0,372757928
PTK	P08069	IGF1R	1,000245329	0,449679352
Type	Kinase Uniprot ID	Kinase Name	Mean Specificity Score	Median Kinase Statistic
STK	Q13627	DYRK1A	2,698970004	0,962910245
STK	P53779	JNK3	2,579060977	0,350300442
STK	P45983	JNK1	2,5456132	0,350300442
STK	P27361	ERK1	2,434502089	0,389524176
STK	P28482	ERK2	2,32536084	0,369860891
STK	P45984	JNK2	2,311478759	0,354757298
STK	O14757	CHK1	2,085766707	0,443626935
STK	Q16539	MAPK14	1,865479292	0,316262218
STK	P06493	CDC2/CDK1	1,808314474	0,321808683
STK	O43293	DAPK3	1,765421042	0,702870446
STK	Q96GD4	AurB/Aur1	1,693159591	0,619502918
STK	O15264	p38[delta]	1,61851957	0,298487534
STK	Q16512	PKN1/PRK1	1,610990368	0,57339075
STK	Q13164	ERK5	1,551132042	0,36437315
STK	P48729	CK1[alpha]	1,469267046	0,359477208
STK	P24941	CDK2	1,344841317	0,294623954
STK	Q9UIK4	DAPK2	1,327745908	0,409874906
STK	O76039	CDKL5	1,237660956	0,411618105
STK	Q00535	CDK5	1,150632344	0,327062551
STK	P50750	CDK9	1,052904009	0,344364034
STK	Q15759	p38[beta]	1,040196203	0,389016528
STK	Q8TD08	ERK7	1,026872146	0,445531862

Table 2. Kinases inhibited by regorafenib treatment in IPAH-PASMCs

Type	Kinase Uniprot ID	Kinase Name	Mean Specificity Score	Median Kinase Statistic
PTK	P06239	Lck	2,172763972	1,662272674
PTK	P00519	Abl	1,890809981	1,429673187
PTK	P07947	Yes	1,611653654	1,55896577
PTK	P42684	Arg	1,33236283	1,374710113
PTK	P51451	BLK	1,048701546	1,497320713
PTK	P43405	Syk	1,01386118	1,238894683
**Type**	**Kinase Uniprot ID**	**Kinase Name**	**Mean Specificity Score**	**Median Kinase Statistic**
STK	Q16512	PKN1/PRK1	2,404383558	9,681785544
STK	P27361	ERK1	1,833963679	4,138932992
STK	O14757	CHK1	1,820464391	5,764893582
STK	P45984	JNK2	1,680841712	4,04511258
STK	P16066	ANP[alpha]	1,564359649	3,910785893
STK	O15264	p38[delta]	1,508065307	3,915147527
STK	P24941	CDK2	1,503467981	4,202821253
STK	P28482	ERK2	1,373572076	3,865930586
STK	P06493	CDC2/CDK1	1,303239493	4,060445271
STK	P53779	JNK3	1,241408003	3,412545259
STK	P45983	JNK1	1,201121141	3,412545259
STK	Q96GD4	AurB/Aur1	1,193538602	5,516586485
STK	Q00535	CDK5	1,138995923	4,47814888
STK	O96017	CHK2	1,096549427	3,597653831
STK	Q96S38	RSKL1	1,060491932	4,608102686
STK	Q16644	MAPKAPK3	1,048248875	3,054903846
STK	O43293	DAPK3	1,016240689	4,562227636

"The “mean specificity score” indicates the specificity of the predicted differential kinase activity with respect to the amount of peptides used for predicting the corresponding kinase. The higher the “mean specificity score”, the less likely it is that the numerical value for the predicted kinase activity could have been generated using a random set of peptides (instead of the original substrates) from the present data set. The “mean specificity score” is calculated by the following equation: -log10 p-value, where $p < 0.05$ refers to the statistical significance for the changes of the phosphorylation for the substrate peptide sets between the two experimental conditions. For kinases with less stringent criteria during the upstream kinase prediction the p-value varies between 0.05 and 1 which results in a mean specificity score between “1 and 1.3."
